# Clinical Evaluation of Edible Oils Used in Traditional Oil Pulling Therapy for Plaque Control and Tooth Discoloration: A Randomized Trial

**DOI:** 10.3390/healthcare14131985

**Published:** 2026-07-03

**Authors:** Ahmet Bedreddin Şahin, Tuğba Aydın

**Affiliations:** Faculty of Dentistry, Department of Periodontology, Atatürk University, Erzurum 25240, Türkiye

**Keywords:** chlorhexidine, oil pulling therapy, dental plaque, plaque regrowth model, tooth discoloration, coconut oil, nigella sativa oil, terebinth oil

## Abstract

**Highlights:**

**What are the main findings?**

- Chlorhexidine achieved significantly greater plaque reduction than coconut, black cumin seed, thyme, and distilled water rinses.
- Oil pulling resulted in significantly less tooth discoloration than chlorhexidine but showed limited plaque-control efficacy.

**What are the implications of the main findings?**

- Oil pulling may be considered an adjunctive oral hygiene practice rather than a replacement for chlorhexidine in plaque control.
- This first controlled clinical comparison of multiple oils provides evidence to guide clinicians and patients regarding the benefits and limitations of oil pulling.

**Abstract:**

**Introduction**: Oil pulling therapy has gained increasing attention as a natural oral hygiene practice; however, evidence regarding its clinical effectiveness remains limited and inconclusive. This study aimed to evaluate the effects of oil pulling therapy on dental plaque regrowth and tooth discoloration compared with chlorhexidine. **Materials and Methods**: One hundred systemically healthy dental students were randomly allocated to five groups: chlorhexidine, coconut oil, black cumin seed oil, terebinth oil, and distilled water. Following professional prophylaxis, participants refrained from mechanical oral hygiene for four days and used their assigned intervention twice daily. Plaque accumulation was assessed using the Turesky modification of the Quigley–Hein Plaque Index, gingival inflammation using the Gingival Index, and tooth color using CIELAB color difference measurements. Data were analyzed using one-way ANOVA or Kruskal–Wallis test with appropriate post hoc tests, depending on the distribution of data. **Results**: Plaque scores differed significantly among groups (*p* < 0.001), with chlorhexidine showing superior plaque inhibition compared with all oil-based interventions and distilled water. Gingival index values were lowest in the chlorhexidine group, although differences among oil groups were not statistically significant. Tooth discoloration was significantly greater with chlorhexidine than with all oil-based interventions (*p* < 0.001). **Conclusions**: Oil pulling therapies demonstrated lower anti-plaque efficacy than chlorhexidine but resulted in less tooth discoloration. These findings suggest that oil pulling may serve as an adjunct rather than an alternative to conventional plaque control.

## 1. Introduction

Dental plaque is a complex microbial biofilm that adheres to oral surfaces and constitutes the primary etiologic factor for gingival and periodontal diseases [1]. Within a few days after plaque accumulation, gingivitis may develop as a localized inflammatory response to bacterial challenge. If left untreated, gingivitis can progress to periodontitis, resulting in attachment loss and alveolar bone destruction [2]. Consequently, effective plaque control remains the cornerstone of preventive dentistry [3,4].

Conventional mechanical plaque control methods, such as toothbrushing and interdental cleaning, are essential but often insufficient to maintain long-term plaque suppression in all individuals [5]. Therefore, adjunctive use of chemical agents has been recommended to enhance oral hygiene efficacy [4,6]. Among these, chlorhexidine (CHX) mouthwash has long been considered the gold standard owing to its broad-spectrum antimicrobial activity and proven efficacy against plaque and gingivitis [6,7,8,9]. However, its long-term use is limited by adverse effects such as tooth staining, taste alteration, and oral mucosal irritation [10,11,12,13]. Furthermore, recent in vitro and clinical evidence has suggested that prolonged CHX exposure may reduce periodontal ligament fibroblast viability, alter the oral pH toward acidity, and decrease nitrate-reducing bacterial populations, potentially impacting oral–systemic balance [13,14,15]. Given the widespread use of over-the-counter oral hygiene products and the growing public interest in natural alternatives, evaluating the clinical effectiveness of such practices is important for evidence-based public health recommendations.

Oil pulling therapy, a traditional oral hygiene practice involving swishing edible oils in the mouth, has been proposed as a natural and biocompatible alternative for chemical plaque control [16,17,18]. Several studies have reported that oil pulling with sesame or coconut oil can reduce plaque and gingival inflammation [16,19,20,21,22,23,24], though the quality and consistency of available evidence remain limited due to small sample sizes, short intervention durations, and methodological bias [25]. Short-term randomized controlled trials have indicated comparable plaque inhibition between oil pulling and 0.2% CHX, with the added advantage of reduced tooth staining [19,21], while others have reported inconsistent or inconclusive findings [20,22,23,24].

Despite increasing interest, few well-designed clinical trials have systematically compared different edible oils under standardized conditions. In particular, the available literature lacks multi-arm randomized controlled studies evaluating multiple oil types within a controlled plaque regrowth model and assessing both clinical and aesthetic outcomes simultaneously. Therefore, the present study was designed as a multi-arm randomized controlled clinical trial to evaluate the effects of selected edible oils—previously reported to possess antimicrobial or anti-plaque properties [26,27,28]—on dental plaque formation and tooth discoloration, in comparison with 0.2% CHX mouthwash.

The primary outcome of this study was plaque inhibition as assessed by the Turesky modification of the Quigley–Hein Plaque Index. Based on the available evidence, we hypothesized that oil pulling with edible oils would inhibit plaque accumulation to a greater extent than distilled water yet would remain inferior to chlorhexidine in terms of antiplaque efficacy.

## 2. Materials and Methods

### 2.1. Study Design, Registration, and Ethical Approval

This single-center, randomized, controlled, observer-blinded, multi-arm clinical trial was designed to evaluate the effect of different oil pulling therapies compared to 0.2% chlorhexidine (CHX) and distilled water (DW) during a four-day plaque regrowth period [19,29,30]. The study was conducted in the Department of Periodontology, Faculty of Dentistry, Atatürk University (Erzurum, Türkiye) between October 2021 and January 2022.

Ethical approval was obtained from the Atatürk University Faculty of Medicine Clinical Research Ethics Committee (Issue No: B.30.2.ATA.0.01.00/412; 5 October 2021), and all procedures were conducted in accordance with the Declaration of Helsinki. Written informed consent was obtained from all participants prior to enrollment. The trial was retrospectively registered at ClinicalTrials.gov (NCT05304338) on 22 March 2022, following study initiation in October 2021, owing to administrative disruptions during the COVID-19 pandemic and delayed recognition of prospective trial registration requirements. The study adhered to the CONSORT guidelines [31].

### 2.2. Study Population

Participants were volunteer dental students from Atatürk University Faculty of Dentistry. Inclusion criteria comprised systemically healthy individuals aged 18–30 years, with ≥24 natural teeth, and without prosthetic or orthodontic appliances or a previous history of periodontitis.

Exclusion criteria included current smoking, pregnancy or breastfeeding, systemic or topical antimicrobial use within the past three months, allergy to study ingredients, ≥2 mm gingival recession, developmental enamel defects or hypomineralization in spectrophotometer-evaluated teeth, and composite restorations on these teeth. Individuals with habits potentially affecting plaque accumulation (e.g., mouth rinsing, gum chewing) were also excluded.

Eligible participants were periodontally healthy, defined by probing depth (PD) < 3 mm, bleeding on probing (BoP) ≤ 10%, absence of clinical attachment loss, and no radiographic evidence of bone loss [32]. Eligibility was confirmed at the end of the two-week pre-experimental phase, during which participants demonstrated adequate oral hygiene compliance and periodontal stability before entering the four-day test period.

### 2.3. Randomization and Blinding

Participants were randomly assigned into five groups (1:1:1:1:1) using a computer-generated randomization list. Product allocation was performed by an independent dentist not involved in data collection or analysis. Clinical examinations and outcome assessments were performed by an examiner who was blinded to group allocation.

To minimize allocation awareness, all mouthwash and oil samples were dispensed in identical opaque 100 mL bottles labeled only with group codes (A–E). Participants were not informed about the content of their assigned product. However, complete participant blinding could not be guaranteed because of differences in taste, smell, texture, and application duration among the study interventions.

### 2.4. Study Groups

- Group A: 0.2% CHX mouthwash (Klorhex^®^; Drogsan, Ankara, Türkiye), alcohol-free formulation, served as the positive control.
- Group B: Cold-pressed coconut oil (CO; Zade Vital, Istanbul, Türkiye).
- Group C: Black cumin seed oil (BCSO; Zade Vital, Istanbul, Türkiye).
- Group D: Terebinth oil (TO; Zade Vital, Istanbul, Türkiye).
- Group E: Distilled water (DW)—negative control.

All products were dispensed in identical opaque bottles. Participants in the CHX and DW groups rinsed with 10 mL of the assigned solution for 30 s twice daily [7,10]. The amount was standardized using 10 mL sterile syringes (Berika, Konya, Türkiye). Oil pulling was performed using one tablespoon (~10 mL) of edible oil swished intraorally for 15–20 min until the oil turned milky white and thin, indicating a change in viscosity. The procedure was performed twice daily, after breakfast and dinner, approximately every 12 h. The visual change in oil consistency was used as a qualitative indicator of correct practice [17,19,22]. Participants used a timer to monitor the duration, and compliance was reinforced via automated reminder messages sent at 8:00 AM and 8:00 PM. These parameters were consistent with previous protocols [19].

During the study, participants were instructed to refrain from all oral hygiene practices other than their assigned intervention—including toothbrushing, mouth rinses, and chewing gum.

### 2.5. Study Protocol

**Figure 1:**
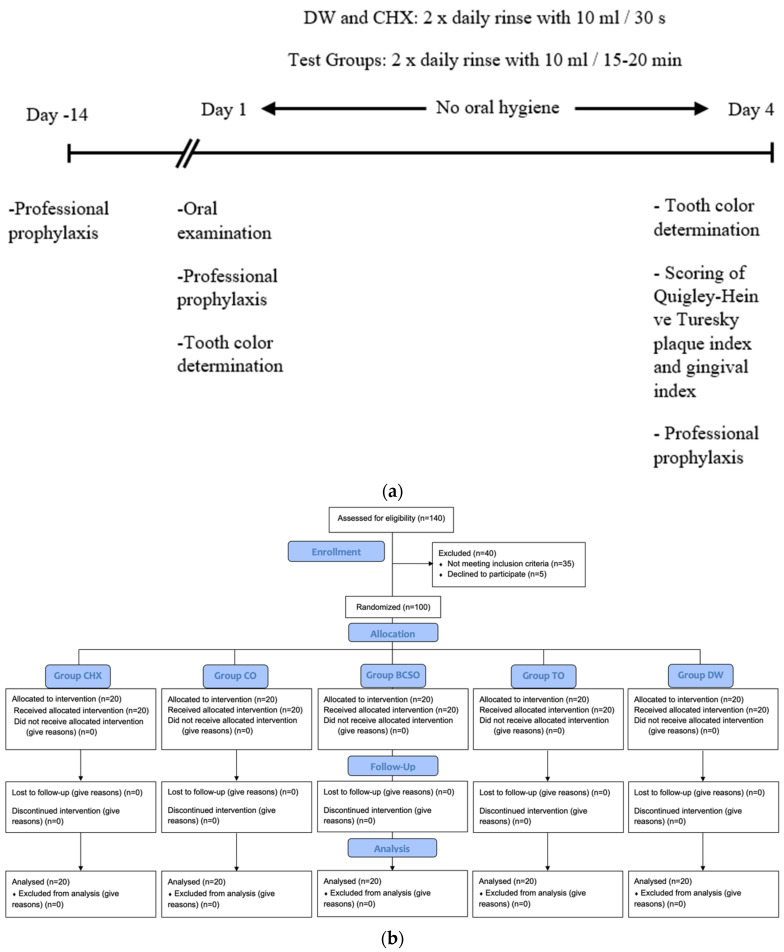
(**a**) Flowchart of the study, (**b**) flow of the patients during the study.

A two-week pre-experimental phase preceded the four-day test period (Figure 1). The two-week pre-experimental phase did not involve the use of any study products; it served exclusively to standardize oral hygiene levels and verify periodontal stability across all participants. All 100 participants who completed the pre-experimental phase were subsequently enrolled in the study, as none were excluded due to non-compliance or insufficient oral hygiene.

At baseline, all participants underwent professional scaling and polishing to eliminate plaque, calculus, and extrinsic stains by a blinded examiner (A.B.S.), ensuring a plaque score of zero. Oral hygiene instruction was provided, and periodontal stability was verified (BoP < 10%) at Day 1 [32].

On Day 1, plaque disclosure (Curaprox, Kriens, Switzerland) was performed to verify plaque-free status. Tooth color was measured using a digital spectrophotometer (VITA Easyshade^®^ V; VITA Zahnfabrik, Bad Säckingen, Germany) by the same examiner. Participants then began using the assigned product under supervision.

On Day 4, participants returned used bottles for compliance assessment. Tooth color was re-measured, followed by plaque and gingival assessments. Plaque accumulation was scored using the Turesky modification (1970) of the Quigley and Hein Plaque Index (QHT) [33,34], and gingival inflammation was evaluated using the Gingival Index (GI) [35].

All clinical measurements were performed by the same blinded examiner. Following data collection, all participants received professional cleaning to remove plaque and stains. Any adverse events or product-related intolerance reported by participants during the study period were recorded.

### 2.6. Clinical Examinations

The QHT Plaque Index quantified plaque accumulation, and the GI measured gingival inflammation. Tooth color measurements were performed with the VITA Easyshade^®^ V spectrophotometer under D65 standard illumination. The middle third of the right maxillary central incisor was selected for color assessment [36,37]. Each measurement was repeated three times, and the mean CIE L*, a*, and b* values were recorded.

The color difference (ΔE) between baseline (L_0_, a_0_, b_0_) and Day 4 (L*, a*, b*) was calculated using the CIELAB formula:

ΔE = [(ΔL)^2^ + (Δa)^2^ + (Δb)^2^]^1/2^, where ΔL = L* − L_0_, Δa = a* − a_0_, Δb = b* − b_0_. (1)

### 2.7. Sample Size Calculation

Based on Sezgin et al. [19], the mean Plaque Index (PI) in the CHX group was 1.61 ± 0.20. A difference of 0.20 units was considered clinically significant. Using G*Power software (version 3.1.9.7; Heinrich Heine University Düsseldorf, Düsseldorf, Germany), a total of 100 participants (20 per group) were required to achieve 80% power at a 95% confidence level. The 0.20-unit difference used in the sample size calculation was derived from the standard deviation of the Plaque Index reported in the CHX group by Sezgin et al. [19] and was considered an appropriate estimate of the minimum clinically meaningful difference under comparable experimental conditions.

### 2.8. Statistical Analysis

All analyses were performed using IBM SPSS Statistics v20.0 (IBM Corp., Chicago, IL, USA). Prior to analysis, the normality of data distribution was assessed using the Shapiro–Wilk test, and homogeneity of variances was evaluated using Levene’s test. For normally distributed variables, group comparisons were conducted using one-way analysis of variance (ANOVA), followed by Tukey’s HSD post hoc test for pairwise comparisons. For non-normally distributed variables, the Kruskal–Wallis test was applied, followed by the relevant post hoc pairwise comparison test. Both post hoc procedures control the family-wise Type I error rate across multiple comparisons. To assess the relationships between continuous variables, Pearson’s correlation was applied for normally distributed data, whereas Spearman’s correlation was used for non-normally distributed data. Effect sizes (eta-squared, η^2^) with 95% confidence intervals were calculated and reported for all outcome variables. Gender distribution among groups was assessed using the chi-square test. All tests were two-tailed, and a *p*-value of <0.05 was considered statistically significant.

## 3. Results

All participants completed the study protocol. No participant discontinued the intervention because of product intolerance or adverse effects during the four-day study period.

No statistically significant difference was found in the gender distribution among the five study groups (χ^2^ = 0.241, *p* = 0.993), indicating that randomization achieved a balanced allocation by sex (Table 1).

### 3.1. Plaque Index (QHT)

A statistically significant difference in mean Plaque Index was found among the groups (H = 47.105; *p* < 0.001), with a large effect size (η^2^ = 0.746; 95% CI: 0.646–0.795). The CHX group had a markedly lower Plaque Index (mean 1.617) than all other groups. Post hoc pairwise comparisons confirmed that the CHX group differed significantly from the BCSO, CO, TO and DW groups, whereas these latter four groups did not differ significantly from one another (Table 2).

### 3.2. Gingival Index (GI)

The mean Gingival Index differed significantly among the groups (H = 14.097; *p* = 0.007), although the effect size was small (η^2^ = 0.148; 95% CI: 0.019–0.251). In post hoc pairwise comparisons, a significant difference was observed only between the CHX group (lowest value, mean 0.1151) and the DW group (highest value, mean 0.1537); the remaining pairwise comparisons were not statistically significant (Table 3).

### 3.3. Tooth Color Change (ΔE)

A highly significant difference in ΔE (color change) was found among the groups (F = 85.693; *p* < 0.001), with a large effect size (η^2^ = 0.783; 95% CI: 0.696–0.825). The CHX group showed the greatest color change (mean 3.782), clearly higher than all other groups. Post hoc pairwise comparisons were significant for BCSO–TO, BCSO–CHX, CO–CHX, CO–DW, TO–CHX and CHX–DW. These findings indicate that the marked discoloration was driven mainly by the CHX group (Table 4).

### 3.4. Summary of Intergroup Comparisons

Plaque Index (QHT): CHX < all oils ≈ DW (*p* < 0.001).

Gingival Index (GI): CHX < DW (*p* = 0.007); no significant differences among oil-pulling groups (*p* > 0.05).

Tooth Color Change (ΔE): CHX > all oils ≈ DW (*p* < 0.001).

### 3.5. Correlation Analysis

Spearman’s rho correlation analysis was conducted to examine the associations among the Plaque Index (PI), gingival index (GI), and tooth color change (ΔE).

A significant positive correlation was observed between PI and GI (r = 0.338, *p* = 0.001) (Table 5).

Conversely, ΔE demonstrated significant negative correlations with both PI (r = −0.427, *p* < 0.001) and GI (r = −0.297, *p* = 0.003). The observed correlations are relationships that emerge when the groups are considered together and largely reflect a treatment-group effect, particularly that of the chlorhexidine group; therefore, they should not be interpreted as causal or direct biological relationships.

## 4. Discussion

The present research was designed to evaluate the efficacy of oil pulling therapy in controlling plaque formation and tooth discoloration in comparison to CHX-containing mouthwash. The results revealed that oil pulling therapy did not achieve the same level of plaque reduction as CHX mouthwash.

The prevention of periodontal diseases depends on the prevention of gingivitis by implementing supragingival plaque control effectively [1]. While mechanical methods, like toothbrushing and flossing, remain fundamental to daily oral hygiene, they may not be sufficient for all individuals. Thus, the XI European Workshop in Periodontology (2014) recommends considering the incorporation of chemical agents when additional plaque control is required [4,38]. CHX has been widely accepted as the “gold standard” of antimicrobial mouthwashes [7,39]. However, long-term use of CHX in daily oral hygiene practices can result in side effects such as tooth discoloration, taste alterations, and mucosal erosions [10]. To address these issues, CHX mouthwashes with low concentrations (0.05%/0.06%) or different formulations (such as CHX with anti-discoloration system) have been developed and are currently available for long-term daily use [7,11]. Furthermore, a study conducted by Guerra et al. showed that the CHX with anti-discoloration system had limited efficacy in reducing dental plaque and gingival bleeding [11]. Thus, the search for an appropriate chemical agent that is safe for long-term use is still ongoing [19,40]. Oil pulling therapy emerged as an alternative method of plaque control [16,19]. Due to the recent growing interest in alternative medicine, oil pulling has gained popularity, as the product and practices are considered natural and safe [20]. Several studies have reported the effectiveness of oil pulling therapy in plaque control, making it a potential alternative to CHX mouthwash [16,19,21].

Despite the unclear effect mechanism of oil pulling therapy, some theories put forward that the saponification and emulsification processes of the edible oils enhance their cleansing effect, while the viscous nature of the oil may inhibit plaque formation and bacterial adhesion [22,41]. Another theory suggests that the antimicrobial properties of the oils used in oil pulling therapy help to destroy microorganisms and control plaque [18,23]. Antimicrobial agents have the ability to prevent bacterial adhesion, colonization, and ultimately metabolic activity that affects bacterial growth [42]. The antimicrobial effects of CO, BCSO, and TO that can be used in oil pulling therapy have been reported in various in vitro studies [26,27]. Based on their reported antimicrobial properties, coconut oil, black cumin seed oil, and terebinth oil were selected as the test interventions in the present study.

CHX is a widely researched and effective agent for plaque control, with various formulations used in dental practice. It is also recognized as a positive control by the pharmaceutical industry for evaluating the efficacy of other anti-plaque agents [8]. Therefore, CHX was chosen as the positive control in this study.

A four-day plaque growth model was chosen for this study, as it has been used for initial screening and typical evaluation of new chemical agents [29]. Plaque growth studies are sufficient to evaluate anti-plaque efficacy and the early occurrence of side effects [30]. The four-day plaque growth model measures plaque regrowth under the influence of the test solution from an initial plaque score of zero and avoids the confounding effect of brushing, which can vary widely among individuals. Hence, it is claimed that this study model provides information about the maximum anti-plaque effect that can be obtained by an agent [19,29].

To the best of our knowledge, our current research is the first to evaluate the clinical efficacy of oil pulling therapy with BCSO and TO on dental plaque. Previous studies have evaluated the effect of oil pulling therapy with coconut oil (CO) on plaque inhibition and reported positive results [19,23]. Additionally, studies using different edible oils such as sesame oil, olive oil, and sunflower oil have also reported the effectiveness of oil pulling therapy in inhibiting plaque [16,21,24]. Contrary to these studies, in our current research, the plaque inhibition efficacy of oil pulling therapy was not found to be positive. In all test groups, QHT Plaque Index scores were found to be statistically higher in comparison to the CHX group. While the results of previous studies were promising, these results cannot be directly compared to the results of the current study because of the different study designs and durations, study models, study populations, and different mouthwash regimens. These heterogeneities may explain the differences in the results.

To our knowledge, there is only one published plaque growth study comparing oil pulling therapy to CHX. Sezgin et al. [19] reported no statistically significant difference between the control and test groups in terms of the GI scores and BoP. In the current research, similar to Sezgin et al., no significant difference was found in terms of GI scores between the test groups treated with oil pulling (CO and BCSO) therapy and the positive control group using CHX. In contrast, a significant difference was found between the test group that used TO for pulling and the positive control group. Only mild inflammation, slight discoloration, or mild edema was detected on visual examination in the tooth regions where the GI score was 1. The clinical relevance of the statistically significant difference in GI scores between the terebinth oil and chlorhexidine groups warrants careful consideration, given that no bleeding on probing was detected in either group and that the transition between GI scores of 0 and 1 is inherently subjective, potentially limiting the clinical significance of this finding.

Different metrics such as the CIELab color difference formula or the CIEDE2000 formula, which aim to improve the verification between calculated and perceived color differences, can be used for the objective determination of color changes [36]. In order to objectively evaluate the color differences in the current study, the CIELAB color difference formula was employed, which has also been utilized in many previous studies [11,37,43]. In this study, the color difference measured in the test groups and the negative control group was found to be lower than that in the positive control group. Ruyter et al. [44] reported the acceptable ΔE value to be ≤3.3. Our evaluations revealed the average ΔE value for the positive control group, which used CHX mouthwash, to be 3.78, which is above the acceptable limit. The average ΔE values for the test groups and the negative control group are within acceptable limits. In addition, the L_0_, a_0_, and b_0_ values representing the color values measured at the beginning and the L*, a* and b* values representing the color values measured at the end of four days revealed that the color differences in all study groups were darker and more yellow in almost all participants. To our knowledge, only one study has compared oil pulling therapy with CHX in terms of tooth discoloration [19]. Sezgin et al. reported that oil pulling therapy caused less tooth discoloration than CHX, consistent with our current study. However, a statistically significant color difference was found between TO and BCSO. The possible reason for this may be that TO contains phenolic substances such as gallic acid and tannic acid, which have been reported to cause tooth discoloration [12,13].

The current study has some limitations. One of these limitations is that the four-day plaque growth model used is insufficient to assess both the antigingivitis effect of oil pulling and its long-term results. However, the purpose of the study was to compare the anti-plaque activity of oil pulling and 0.2% CHX mouthwash. This study design, as stated in the literature, is appropriate to provide information about the maximum anti-plaque effect that can be obtained by an agent [19,29]. To minimize information bias, measures were taken to ensure that the individuals responsible for product allocation and recording clinical parameters were different in the study. Additionally, to reduce the potential for selection bias, all the participants in test and control groups are composed of voluntary students who study in the Faculty of Dentistry at Ataturk University. Furthermore, there was a significant difference in the duration of product use between the control and test groups, which may have affected participants’ compliance with product usage. To address this issue, compliance was monitored through the first use of the allocated product and the collection of the used product. In addition, the right duration of use of the products was reminded twice a day by the dentist who allocated the products. Another limitation of the study was that while the study attempted to blind participants, differences in taste, color, and smell of the test products may have compromised blinding. This could have introduced bias if participants guessed their group allocation, potentially influencing their behavior or reporting. Formal intra-examiner calibration and reliability assessment were not performed for the Plaque Index and Gingival Index measurements prior to data collection, which may be considered a methodological limitation. Future studies should incorporate standardized calibration exercises and report reliability coefficients to strengthen the reproducibility of clinical measurements. In addition, color measurements were restricted to the right maxillary central incisor, which is the most commonly used reference tooth in spectrophotometric dental research [36,37]. Since the distribution of rinsing products within the oral cavity may vary among participants, discoloration patterns on other teeth may differ, which should be considered a limitation of the present study. Furthermore, color measurements were reported as ΔE values only; individual L*, a*, and b* components were not separately analyzed, which limits the interpretation of the direction of color change across groups.

According to the findings of our study and within the limitations, it appears that oil pulling therapy does not provide the claimed anti-plaque activity. However, it is important to note that the mechanism of action of agents used in plaque control may vary, such as their anti-plaque and anti-gingivitis effects [6,38]. In many studies, chemical agents that work by antiadhesion, “chemical” removal, or effects on the biofilm matrix are typically tested, while agents with different mechanisms of action are often overlooked [9]. Oil pulling therapy may provide benefits to plaque control through different mechanisms of action, such as reducing the number of microorganisms in the biofilm or altering its pathogenicity [18,38,41]. The fact that the participants in the test group had similar GI values to those in the CHX group may also support this conclusion. However, it is important to note that the clinical symptoms of gingivitis can be seen in the early lesion stage after the fourth day [45]. This may also be why GI scores were observed similarly with no BoP in the study participants. It should be noted that in this study, participants refrained from all mechanical oral hygiene for four days, which differs from real-world practice, where oil pulling is used alongside regular toothbrushing rather than as a replacement. Therefore, the anti-plaque findings of this study should be interpreted within the context of this experimental model and may not directly reflect the outcomes expected in routine clinical use.

The study has employed a specialized population to provide homogeneity that consisted solely of young, healthy dental students, which limits the generalizability of the results. This group may not represent the general population, especially those with existing oral health issues or different demographics. Different study designs and further studies with larger samples are needed to fully shed light on whether oil pulling therapy is effective in plaque control and its mechanism.

## 5. Conclusions

Within the limitations of this randomized controlled clinical trial and in the context of a short-term, four-day plaque regrowth model, oil pulling therapies demonstrated lower anti-plaque efficacy compared with chlorhexidine, although they resulted in significantly less tooth discoloration. These findings suggest that oil pulling may be considered as an adjunct rather than a substitute for conventional chemical plaque control. Given the increasing use of natural oral hygiene practices, further well-designed long-term clinical studies are needed to better understand their effectiveness and mechanisms of action.

## Figures and Tables

**Table 1:** Distribution of participants by gender among study groups.

Gender	BCSO (n, %)	CO (n, %)	TO (n, %)	CHX (n, %)	DW (n, %)	χ^2^	*p*
Female	9 (45.0%)	10 (50.0%)	9 (45.0%)	10 (50.0%)	9 (45.0%)	0.241	0.993
Male	11 (55.0%)	10 (50.0%)	11 (55.0%)	10 (50.0%)	11 (55.0%)		

**Table 2:** Plaque Index (QHT) scores after four days of plaque regrowth.

Group	Mean (95% CI) ± SD	Median (Min–Max)	Effect Size (95% CI)	Test	*p*	Post hoc
BCSO	2.877 (2.767–2.987) ± 0.235	2.929 (2.214–3.125)	η^2^ = 0.746 (95% CI: 0.646–0.795)	H = 47.105	<0.001	CHX vs. all other groups
CO	2.921 (2.719–3.123) ± 0.431	2.929 (1.509–3.375)				
TO	2.987 (2.901–3.074) ± 0.186	2.991 (2.589–3.321)				
CHX	1.617 (1.406–1.828) ± 0.451	1.750 (0.946–2.179)				
DW	2.968 (2.896–3.039) ± 0.153	2.946 (2.696–3.232)				

Mean (95% CI) ± SD: Mean (95% confidence interval) ± standard deviation; Median (Min–Max). Effect size is eta-squared (η^2^). Kruskal–Wallis test with post hoc pairwise comparisons was applied.

**Table 3:** Gingival Index (GI) values after four days of plaque regrowth.

Group	Mean (95% CI) ± SD	Median (Min–Max)	Effect Size (95% CI)	Test	*p*	Post hoc
BCSO	0.1442 (0.1245–0.1638) ± 0.0420	0.1428 (0.0714–0.2321)	η^2^ = 0.148 (95% CI: 0.019–0.251)	H = 14.097	0.007	CHX vs. DW
CO	0.1267 (0.1146–0.1388) ± 0.0258	0.1250 (0.0714–0.1696)				
TO	0.1490 (0.1277–0.1703) ± 0.0455	0.1473 (0.0803–0.2410)				
CHX	0.1151 (0.1002–0.1300) ± 0.0319	0.1071 (0.0714–0.1696)				
DW	0.1537 (0.1398–0.1675) ± 0.0295	0.1516 (0.1147–0.2131)				

Mean (95% CI) ± SD: Mean (95% confidence interval) ± standard deviation; Median (Min–Max). Effect size is eta-squared (η^2^). Kruskal–Wallis test with post-hoc pairwise comparisons was applied.

**Table 4:** Mean tooth color change (ΔE) values after four days.

Group	Mean (95% CI) ± SD	Median (Min–Max)	Effect Size (95% CI)	Test	*p*	Tukey’s HSD Test
BCSO	1.326 (1.110–1.541) ± 0.461	1.145 (0.728–2.410)	η^2^ = 0.783 (95% CI: 0.696–0.825)	F = 85.693	<0.001	BCSO–TO; BCSO–CHX; CO–CHX; CO–DW; TO–CHX; CHX–DW
CO	1.474 (1.253–1.695) ± 0.472	1.454 (0.300–2.427)				
TO	1.702 (1.553–1.851) ± 0.318	1.650 (1.118–2.385)				
CHX	3.782 (3.420–4.144) ± 0.773	3.752 (2.307–5.420)				
DW	1.490 (1.338–1.641) ± 0.324	1.510 (1.025–2.377)				

Mean (95% CI) ± SD: Mean (95% confidence interval) ± standard deviation; Median (Min–Max). Effect size is eta-squared (η^2^). F: One-way ANOVA with Tukey’s HSD test was applied. The CHX group showed the highest ΔE value.

**Table 5:** Spearman’s rho correlation coefficients among clinical parameters.

Variables	Plaque Index	Gingival Index	ΔE
Plaque Index	1	0.338 **	−0.427 **
Gingival Index	0.338 **	1	−0.297 **
ΔE	−0.427 **	−0.297 **	1

** Correlation is significant at the 0.01 level (2-tailed).

## Data Availability

The data presented in this study are openly available in Zenodo at https://doi.org/10.5281/zenodo.18495493.
